# Development of Sodium Alginate Bioplastic Reinforced with Dried Orange Juice By-Product for Use in Packaging

**DOI:** 10.3390/polym16233382

**Published:** 2024-11-30

**Authors:** Pedro H. S. Bezerra, Yves J. Souza-Santos, Eliria M. J. A. Pallone, Rosemary A. Carvalho, Fernanda M. Vanin

**Affiliations:** 1 Food Engineering Department, Faculdade de Zootecnia e Engenharia de Alimentos (USP/FZEA), Multiuser Center for Macromolecule Functionality (CEMFUM), Universidade de São Paulo, Av. Duque de Caxias Norte 225, Pirassununga 13635-900, SP, Brazil; pedrobezerra315@usp.br (P.H.S.B.);; 2 Biosystems Engineering Department, Faculdade de Zootecnia e Engenharia de Alimentos (USP/FZEA), Multiusuário de Caracterização de Materiais (MULTMAT), Universidade de São Paulo, Av. Duque de Caxias Norte 225, Pirassununga 13635-900, SP, Brazil; eliria@usp.br

**Keywords:** *Citrus sinensis*, biopolymers, circular economy

## Abstract

Pollution caused by nonrenewable plastics has driven the use of natural polymers. Similarly, the disposal of food waste still harms the environment. Considering both aspects, this study aimed to evaluate the effect of incorporating orange by-product powder (OBP) as a reinforcing material into sodium alginate films with glycerol. Sodium alginate-based films were produced using glycerol and various concentrations of OBP. The films were characterized in terms of thickness, color, water content, mechanical properties, light transmission, transparency, X-ray diffraction (XRD), Fourier-transform infrared spectrometry (FTIR), contact angle, solubility, swelling, scanning electron microscopy (SEM), and thermogravimetric analysis (TGA). The addition of OBP significantly (*p* < 0.05) reduced the water content of the film from 37.75% ± 5.80a (0-OBP) to 24.49% ± 1.47b (45-OBP). The higher the concentration of OBP, the higher the tensile strength of the films, from 7.99 MPa ± 0.91a (0-OBP) to 18 MPa ± 1.38d (45-OBP), and the higher the hydrophobicity, from 57.60° ± 0.41a (0-OBP) to 70.34° ± 0.98c (45-OBP). From TGA and XRD analyses, it was observed that the incorporation of OBP resulted in less crystalline and more thermally resistant materials. Therefore, this study shows that OBP is a promising reinforcing component for sodium alginate films.

## 1. Introduction

Environmental impact is a growing challenge in different product development chains. These aspects have been widely discussed in recent years in line with the Sustainable Development Goals (SDGs) established under the coordination of the United Nations, which aim to guarantee, by 2030, a more prosperous, equitable, and healthy planet [1]. Therefore, the search for substitutes that meet the need for fossil-based polymers for packaging production that do not cause environmental imbalance has been widely undertaken in recent decades [2]. According to European Bioplastics [3], the production of natural polymers and biopolymers currently represents approximately 0.5% of the total annual plastic output; however, it is estimated that, by 2028, this market could evolve to 1.8% of the annual production. Similarly, the elevated levels of waste related to producing fruits and other vegetables, which account for 28% of food waste, represents one of the challenges of the coming years [4].

Sodium alginate is a popular natural polymer that is widely used owing to its biocompatibility, uniform gelation properties [5], controlled release of coating, release systems, and food packaging applications [6,7]. However, biofilms produced with sodium alginate have low hydrophobicity and poor mechanical properties, requiring the insertion of reinforcing components into the polymeric matrix [8]. Some studies have sought to add reinforcing components such as pectin [9], cellulose [10], and carboxymethylcellulose [11] to enhance the properties of sodium alginate films. Moreover, the use of industrial waste or by-products as reinforcing components in the production of sodium alginate-based films has been explored in recent years [12,13,14].

Lu et al. [12] verified the effects of incorporating peach peel into alginate-based films. According to the authors, the films incorporated with peach peel waste showed better responses in terms of structural properties, such as water resistance and greater hydrophobicity compared to the control film. Hu et al. [13] evaluated the addition of coffee husks to a chitosan and sodium alginate matrix to create an active packaging that protects meat products. Increasing the amount of coffee husks improved the light barrier properties compared to the control film, contributing to the preservation of meat products. Santos et al. [14] investigated the production of sodium alginate films enriched with red onion peel extract. The addition of the extract increased the water solubility of the films, making them more hydrophobic because sodium alginate has a high affinity for aqueous environments.

Therefore, these studies highlight the potential use of by-products, including oranges, as reinforcement materials to produce films.

Brazil is one of the world’s largest producers of orange [15] and generates a significant amount of waste. In addition, oranges are rich in important compounds, such as pectin, which reinforces their value for application [16,17,18]. He et al. [19] used pectin with sodium alginate containing tannins and Fe^+3^ ions to coat passion fruits and increase their shelf life. The authors reported that the use of pectin in films improved the mechanical properties owing to the better cross-linking of the structural components. In addition, the study reported by Qiu et al. [20] produced sodium alginate films with pectin from apricot peel and anthocyanins for the preservation of meat products. Sodium alginate and pectin films showed better cross-linking than those with added anthocyanins. Therefore, these studies underlines the possible use of by-product fruits, such as pectin, to improve film properties [19,20].

However, even when using a by-product from orange, or other fruit processes, it should be underlined that those strategies still generate waste/by-products, as only part of the waste was used. Consequently, a high volume of waste material is still generated and disposed of, which causes environmental impact problems. The full application of this by-product as reinforcement in alginate films is justified, as it promotes the circular economy of this by-product [21].

Therefore, this study aimed to evaluate the impact of incorporating the total by-products from orange juice as a reinforcing material in sodium alginate-based films. In this context, it must be noted that this strategy is aligned with the 12th Sustainable Development Goal (SDG) of the United Nations (UN), which aims for responsible consumption and production as well as a sustainable route aligned with the definitions of the circular economy aimed at the full use of the orange juice by-product in the production of biodegradable materials.

## 2. Materials and Methods

### 2.1. Materials

The films were produced using sodium alginate acquired from êÊxodo Científica (Sumaré, Brazil) and glycerol purchased from Dinâmica (São Paulo, Brazil). The by-product of orange juice production used for film production was donated by local commerce (Pirassununga, Brazil).

### 2.2. Methods

#### 2.2.1. Orange By-Product Powder (OBP)

Orange by-product powder (OBP) was produced according to Merino and Athanassiou [22] with some modifications. The by-products of orange juice production, including peel, albedo, seeds, and membranes, were sanitized using a sanitizing solution (water and 2% sodium hypochlorite). Afterwards, the material was cut into strips and dried in an air circulation oven at 40 for 48 h (Marconi, MA036, Piracicaba, Brazil), followed by milling (Marconi, MA090, Piracicaba, Brazil), particle size standardization (120 mesh), and then storage under refrigeration (−22 °C).

#### 2.2.2. Production of Films Based on Sodium Alginate

Sodium alginate (SA) film was produced using the methodology described by Sucheta et al. [17], with modifications. To define SA and glycerol concentrations, preliminary tests were performed, and the film properties were evaluated in relation to the mechanical properties (tensile strength and elongation). Firstly, SA concentration in films varied from 0.5 g to 2.5 g, using 2.5 g of glycerol and 2.5 g of OBP (Figure S1). Subsequently, different concentrations of glycerol (from 20% to 50%) were evaluated, fixing the SA and OBP concentrations at 2.5 g for both (Figure S2). Considering these results, the SA and glycerol concentrations were fixed. Therefore, films were produced using solution (SA) (1.0 g/100 g film-forming solution), glycerol (1.75 g/100 g film-forming solution), and OBP at the following concentrations: 0-OBP (control, 0 g/10 g SA), 25-OBP (25 g/10 g SA), 35-OBP (35 g/10 g SA), and 45-OBP (45 g/10 g SA).

First, OBP and glycerol were added to distilled water and maintained at 70 °C in a thermostatic bath (Marconi, MA127/GT60, Piracicaba, Brazil). Sodium alginate was added and allowed to completely dissolve by mechanical agitation (IKA-RW20 DIGITAL, Staufen, Germany) at 500 rpm. The mixture was heated in an ultra-thermostatic bath at 95 °C and stirred at 300 rpm for 30 min. Subsequently, the film solution underwent the degassing process formed in the previous stage using ultrasound (Ultronique, Q3.0/40ª, Indaiatuba, Brazil) for 30 min. The film solution was then spread using an automatic spreader (Zehntner ZAA2300, Sissach, Switzerland) to standardize its thickness. Finally, the films were placed in a forced-air circulation oven (Marconi, MA036, Piracicaba, Brazil) at 60 °C for 4 h. The OBP film samples were preconditioned according to ASTM D618-21 in desiccators containing a sodium bromide solution (relative humidity 50 ± 2%; temperature 25 ± 2 °C) [23]. Each formulation was produced in three independent batches, and from each batch, three samples were analyzed for each proposed analysis, giving a total of nine samples for each formulation for each proposed analysis. Therefore, all reported mean values are based on these nine independent measurements.

#### 2.2.3. Film Characterization

##### Thickness

Film thickness was measured using a digital caliper (Mitutoyo, ID-C112XB, Kawasaki Minato City, Tokyo, Japan) at ten random points.

##### Color Measurements

Color analysis was conducted following the method outlined by Gennadios et al. [24], using a HunterLab colorimeter (Miniscan XE, HunterLab, Reston, VA, USA). The color parameters, L*, a*, and b*, of the outer surfaces were measured. The overall color difference (ΔE) was calculated using Equation (1):

(1) $$\Delta E=\sqrt{{\left(L^{*}-L^{0}\right)}^{2}+{\left(a^{*}-a^{0}\right)}^{2}+{\left(b^{*}-b^{0}\right)}^{2}}$$

Each experiment was performed in triplicate. $L^{0}$, $a^{0}$, and $b^{0}$ are the control standards (0-OBP).

##### Water Content

The water content was determined according to the AOAC (Association of Official Analytical Chemists) method [25]. Film samples (2 g) were placed in containers (weight filters) and weighed again (W0). The system (film + weigh-filters) was placed in an oven at 105 °C for 24 h (Fanem, Modelo 515, Guarulhos, Brazil), and after this time weighed again (W1). After this period, the water content was calculated using Equation (2).

(2) $$\mathrm{Water}\;\mathrm{content}=\frac{w0-w1}{w0}\times 100\%$$

##### Mechanical Properties

The mechanical properties were measured to evaluate the tensile strength, elongation, and Young’s modulus of the films, as determined by tensile tests using a TA.XT Plus texturometer (TA Instruments, New Castle, DE, USA), following the methodology described in ASTM D882-10 [26]. The analysis conditions were as follows: initial distance between the probes, 8 cm; constant test speed, 1.0 mm/s, until the films ruptured.

##### Light Transmission and Transparency

The barrier properties in the UV/Vis region were determined using a spectrophotometer (Perkin Elmer, Lambda 35, Waltham, MA, USA). Samples of the films were sized (5 cm × 1.5 cm) and scanned in the wavelength range of 200–800 nm, following the methodology described by Fang et al. [27]. The transparency of the samples was calculated by Han and Floros [28] using Equation (3):

(3) $$\mathrm{Transparency}\;(\%)=\frac{Abs_{600}}{T}$$

where $Abs_{600}$ is the absorbance at 600 nm and T is the thickness of the sample.

##### Fourier-Transform Infrared (FTIR) Spectroscopy

Spectral data of SA-based films with different concentrations of OBP were obtained using Fourier-transform infrared spectroscopy (Spectrum One spectrophotometer, Perkin-Elmer, Waltham, MA, USA) with a coupled accessory to measure attenuated total reflectance (ATR). The analyses were carried out using a resolution of 32 scans carried out at 4 cm^−1^ in the 500–4000 cm^−1^ spectral range. Deconvolution was performed to verify the areas of the -OH bond peaks (near 3285 cm^−1^) based on the Gaussian model applied to normalized spectra using OriginLab (version 2019b) (Northampton, MA, USA) (Figure S3).

##### X-Ray Diffraction

X-ray diffraction (XRD) analyses were performed using a Rigaku Miniflex 600 X-ray diffractometer (Tokyo, Japan). The instrument was operated at 40 kV and a current of 15 mA. The diffractograms were recorded at room temperature and the analysis was conducted over an angle range of 2–50° at a scanning speed of 2°/min.

##### Contact Angle (CA)

The analysis was performed by placing a drop of water in contact with the surface of the film, using an Optical Tensiometer (KSV Instruments, Biolin Scientific AB, Gothenburg, Sweden). The films were analyzed using images taken every second for 10 s. Each image generated by the angle formed between the surface of the biopolymer and the tangent of the water drop was determined using software integrated into the equipment [29].

##### Solubility

Water solubility analysis was performed according to the method described by GONTARD et al. [30]. Disk-shaped samples (2 cm) were immersed in 50 mL of distilled water with mechanical stirring for 24 h at 25 °C. The samples were then dried in an oven for 24 h at 105 °C to measure the final dry mass.

##### Swelling

The swelling index of the 1 cm^2^ films was calculated following the methodology described by Cabello et al. [31]. First, the films were dried in a vacuum oven (Marconi, MA030, Piracicaba, Brazil) at 50 °C for 24 h, and then weighed to obtain the dry mass ($W_{d}$). Next, the dried film was immersed in 50 mL of distilled water at 25 °C for 12 h. After removing the excess water, the film was reweighed to obtain the wet mass ($W_{w}$). The swelling index was calculated using Equation (4).

(4) $$\%\mathrm{SI}=100\;\frac{W_{w}-W_{d}}{W_{d}}$$

##### Scanning Electron Microscopy (SEM)

The surfaces and cross-sections of the films were analyzed using a scanning electron microscope (Hitachi TM-3000 microscope, Hitachi, Tokyo, Japan) with an acceleration voltage of 5 kV.

##### Thermogravimetric Analysis (TGA)

The samples (10–15 mg) were heated in a thermogravimetric apparatus (NETZCSH model STA 440 F3 Júpiter, Selb, Germany) from 25 to 600 °C in a nitrogen atmosphere (100 mL/min) and from 600 to 900 °C in oxygen (100 mL/min). The heating rate was set to 20 °C/min [32].

##### Statistical Analysis

Analysis of variance (ANOVA) and Tukey’s test were used to assess the differences between means. Statistical analysis was performed using SAS software (version 9.4) with a significance level of *p* < 0.05.

## 3. Results and Discussion

### 3.1. Thickness

Analyzing the film thickness is a fundamental part of developing new products, as it relates to other properties, such as mechanical, optical, and thermal properties. The reinforcement of SA-based films with OBP significantly increased their thickness (Table 1). However, increasing the OBP concentration did not significantly affect the film thickness.

Films formulated without OBP exhibited significantly lower thicknesses than films containing OBP (*p* < 0.05) (Table 1). However, the increase in OBP concentration did not significantly affect the film thickness.

A similar finding was reported by Kevij et al. [33]. The authors observed that for films produced with gelatin films and orange peel powder at different concentrations (3–15%), lower concentrations of orange peel powder resulted in a slight increase in the thickness of the films. At higher orange peel powder concentrations, the thickness of the films increased by up to 21%. This increase was attributed to the higher solid content of the gelatin films, as orange peel contains soluble and insoluble fibers that do not dissolve completely, resulting in a thicker structure [34].

In comparison, another study analyzing by-products, carried out by Grisales-Mejía et al. [35] with avocado seed-based films, observed similar results, although the thickness of the films was greater than that in this study, ranging from 0.087 to 0.118 mm. This difference may be associated with the interaction between the biopolymer and by-product, which influences the formation and final thickness of the film.

### 3.2. Color Measurements

The color parameters of the SA-based films were significantly affected by the incorporation of OBP and its concentration. The higher the concentration of OBP, the lower the values of L* and the higher the values of a* and b*. The increase in a* and b* values has been attributed to the polyphenols and carotenoids present in OBP. Kevij et al. [33] produced orange peel films with fish gelatin, and when evaluating their colorimetric characteristics, they observed an increase in a* values, attributing this effect to the polyphenols present in the orange peel. Similarly, Yun and Liu [36] produced mandarin peel films with sodium alginate and reported an increase in b* values, relating this effect to the levels of carotenoids and polyphenols present in the peel. The color difference (ΔE) of the sodium alginate films also significantly increased with the addition of OBP. This change was also observed by Han and Song [37]. The authors produced films with kiwi waste extract and watermelon rind pectin, and observed an increase in ΔE owing to the presence of phenolic compounds and natural pigments.

When compared with other by-products, as in the study by Hanani, Yee, and Nor-Khaizura [38], which evaluated fish gelatin films incorporated with pomegranate peel powder, the values obtained in b* (8.81–0.65) and L* (87.74–72.15) are higher in this research, which represents a better alternative for food preservation as it prevents the oxidative deterioration of food caused by exposure to visible light.

The results expressed are justified by the possible presence of active components, such as polyphenols and carotenoids, in OBP. In addition, the color difference (∆E) increased with the addition of OBP, suggesting that the bioactive compounds present significantly contributed to the change in the colorimetric characteristics of the films, as observed in related research involving other by-products. These findings reinforce the potential of OBP as a color-modifying agent for biopolymer-based materials.

### 3.3. Water Content

The incorporation of OBP into the production of sodium alginate-based films led to a reduction in their water content (Table 1). In general, the water content values presented in this study are similar to those reported by Sucheta et al. [17] for sodium alginate, pectin, and orange peel films, in which the loss of moisture content is related to the ability of the films to form intermolecular interactions between SA and OBP, leaving a film with a lower volume of free water.

However, increasing the OBP concentration did not significantly affect the water content of the films (*p* > 0.05). Castro et al. [39] proposed the production and characterization of OBP under conditions similar to those used in the present study. According to their results, OBP is composed of 73.61 g/100 g (dry matter). Therefore, it could be supposed that the higher levels of fiber in OBP could lead to the development of a “hydrogel” structure in the matrix film, which in turn results in less free volume.

### 3.4. Mechanical Properties

In general, incorporating OBP into SA-based films, as well as increasing the concentration of OBP, led to a significant increase in the tensile strength and Young’s modulus of the films and a significant reduction in elongation at break values (*p* < 0.05) (Table 1). It can be proposed that OBP has an affinity with the sodium alginate matrix and acts as a reinforcement of the mechanical strength owing to components such as pectin. Pectin and sodium alginate, rich in -OH and -COOH groups, form a stable and thermally irreversible three-dimensional network structure by cross-linking with Ca^2+^, Zn^2+^, and Mg^2+^, and because calcium is present in OBP, it ultimately contributes to these bonds [20].

The results obtained in this study corroborate those reported in the literature. In the study proposed by Leites et al. [40], starch extracted from cassava was enriched with an aqueous extract and orange by-product powder. The authors reported an increase in the tensile strength when orange by-product powder was added to starch films. This increase in strength was attributed to the interaction of the by-product fibers with the polymer matrix, which also filled the aqueous spaces, resulting in less water being available in the film. This effect was also observed in this study (in accordance with the results reported for the water content, Section 3.3). Similar results were obtained by Makaremi et al. [41] for sodium alginate films containing pectin at different degrees of cross-linking. The reduction in elongation can be attributed to intermolecular interactions and cross-linking, which make the film stiffer, in addition to the previously discussed aspects of the water content and reduced free volume.

In comparison with other by-products, as demonstrated by Rodsamran and Sothornvit [42] for films using pineapple peel extract, SA-based films with OBP showed better mechanical properties, increasing interest in using this by-product for biofilm production systems.

### 3.5. Light Transmission and Transparency

**Figure 1:**
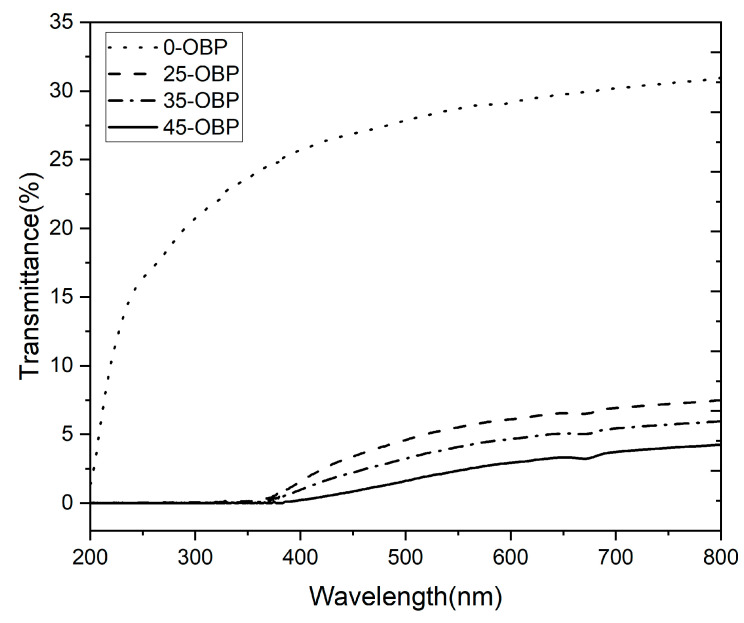
UV–visible spectrum profile of sodium alginate-based films reinforced with different concentrations of OBP (orange by-product): 0-OBP (0 g of OBP); 25-OBP (2.5 g of OBP); 35-OBP (3.5 g of OBP); and 45-OBP (4.5 g of OBP).

Figure 1 shows the light transmission and transparency properties of the films in the UV (200–400 nm) and visible (Vis) regions (400–800 nm). In general, the reinforcement of sodium alginate-based films with OBP reduced their transmittance, indicating improved barrier properties in the UV/Vis region for films reinforced with OBP. Therefore, SA-based films reinforced with OBP showed reduced transmittance in the UV region and could be used for protection against oxidation reactions in food products.

Different subregions can be identified in the UV region: UV-A (320–400 nm), UV-B (280–320 nm), and UV-C (190–290 nm) [43]. None of the OBP film formulations showed bands in the UV-C or UV-B regions. However, in the UV-A range, the 25-OBP and 35-OBP films exhibited slight light transmission.

The end of the 400 nm region marks the beginning of the visible region, which is crucial for the consumer’s perception of the product. A reduction in transmittance could be associated with the presence of essential oils in the OBP. The higher the OBP concentration in the films, the lower the UV transmittance of the SA-based films. When studying edible films produced with carrageenan, trehalose, and orange essential oil, Simona et al. [44] observed that the addition of orange essential oil led to a reduction in UV transmittance in edible films produced with these components. Grisales-Mejía et al. [35] found similar results when avocado residue was used in films, attributing this effect to the cyclic organic molecules present in the avocado residue. Protection in the UV region, that is, a reduction in UV transmittance, is essential for the preservation of the packaged product and the durability of the polymeric material [45].

The transparency of the 0-OBP film was significantly higher (*p* < 0.05) than that of films containing OBP (Table 1). There were no significant variations in the transparency values of the films produced with different concentrations of OBP, which could be attributed to the insoluble components present in the powder, thus reducing the transparency of the films. This phenomenon was observed by Leites et al. [40], who produced films based on cassava starch using orange juice waste, and by Li et al. [46], who produced films from sodium alginate, gelatine, and graphene oxide. The authors reported transparency values close to those of the controls used in this study.

### 3.6. Fourier-Transform Infrared Spectroscopy (FTIR)

**Figure 2:**
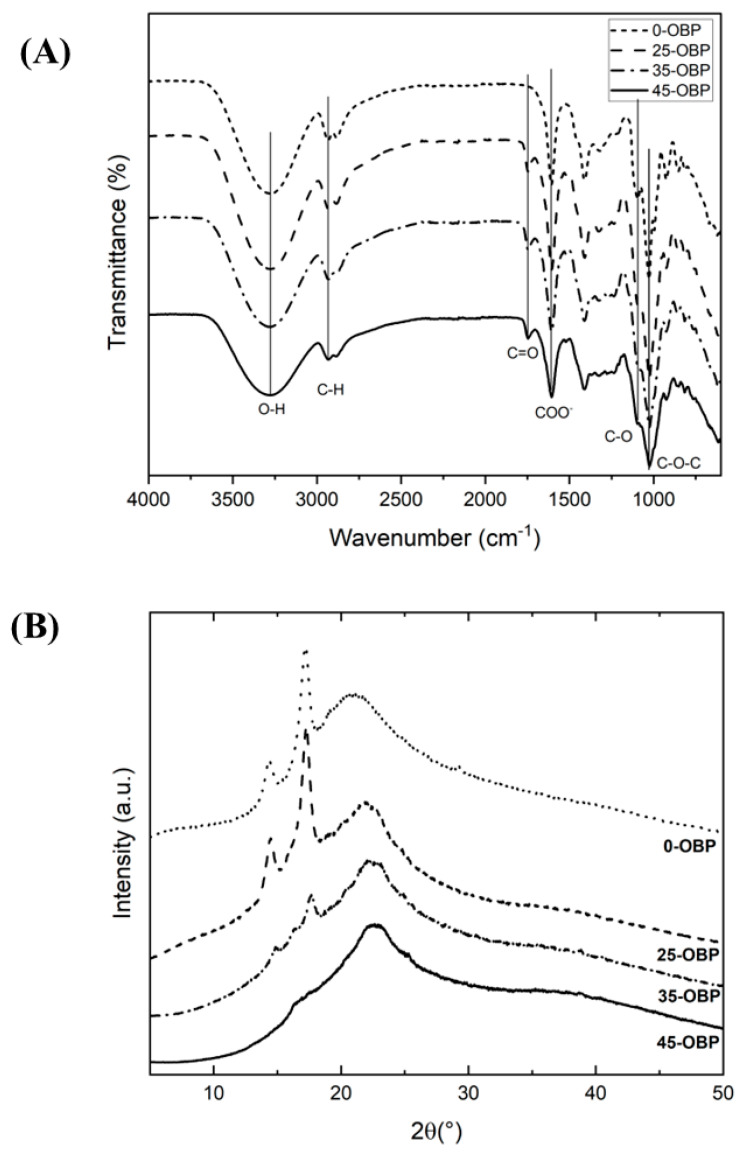
(**A**) FTIR spectra and (**B**) XRD pattern of sodium alginate-based films reinforced with different concentrations of OBP (prange by-product): 0-OBP (0 g of OBP); 25-OBP (2.5 g of OBP); 35-OBP (3.5 g of OBP); and 45-OBP (4.5 g of OBP).

Figure 2A presents the Fourier-transform infrared spectroscopy of the SA-based films reinforced with different concentrations of OBP.

The -OH peaks are broad and less intense between 3000 and 3700 cm^−1^. Therefore, considering this region, it could be observed that the inclusion of OBP did not shift the -OH band to lower wavenumbers; however, the intensity was reduced (Figure 2A). To better elucidate the reactions of the -OH bonds, the FT-IR spectra were deconvoluted into peaks centered at 3285 cm^−1^ (Figure S3). It is evident that as the incorporation of OBP increased, there was a significant reduction in the signal intensity between the 0-OBP and 35-OBP samples, with the intensity of the 45-OBP sample being nearly identical to that of the 35-OBP sample (Figure S3). This reduction could be attributed to greater intermolecular interactions with water molecules and hydrogen bonds between the hydroxyl groups of the polysaccharide fractions [47]. A similar behavior was recently reported for films produced with orange peel–alginate–glycerol [48] and corn starch biofilm reinforced with up to 4 g of orange peel powder [49], while for coal, the bands shifted to lower wavenumbers [50].

The region of the C-H group around 2903 cm^−1^ is correlated with the -CH (saturation) of the glycerol chain [48]. The peak representing the C=O groups of the ester bands appeared at approximately 1700 cm^−1^ due to the carboxylic esters present in the OBP components and was observed only in SA-based films containing OBP; a similar observation was reported by Sánchez-Orozco et al. [48].

The peak around 1605 cm^−1^ corresponds to carboxyl groups. The presence of peaks at approximately 1605 and 2932 cm^−1^ may indicate possible hydrogen bonds between the bioplastic components, which are indicative of the good mechanical properties of the materials produced [12].

The absorption band around 1200 cm^−1^ resulted from the bending of the aliphatic chain, asymmetric carboxyl vibrations, and carboxylic acid stretching of the C-O groups in pectin [51]. The last vibration analyzed was that of the C-O-C group, which showed variations in intensity compared to the 0-OBP control due to the interaction of sodium alginate with the OBP and the presence of aliphatic groups [52].

### 3.7. X-Ray Diffraction (XRD)

X-ray diffraction (XRD) patterns of the SA-based control films (0-OBP) showed a small peak at 15° and a larger peak at 17° (Figure 2B). In the films with added OBP, the crystallinity peaks decreased as the OBP concentration increased, resulting in a more progressive amorphous matrix. Sánchez-Orozco et al. [48] reported a similar effect for sodium alginate films with orange peel powder; the crystallinity decreased compared with the film without orange peel. The authors attributed this effect to the increase in hydrogen bonds owing to the incorporation of orange peel into the alginate matrix with glycerol. A similar effect was observed for SA-based films reinforced with OBP. No characteristic peak was observed in the OBP diffractograms, indicating that the reinforcement material was completely amorphous [53].

### 3.8. Hydrodynamic Properties: Contact Angle, Solubility, and Swelling

The contact angle was used to evaluate the hydrodynamic behavior of the films. The incorporation of OBP into the SA-based films significantly increased (*p* > 0.05) the hydrophobic characteristics of the films (Table 1); however, the films continued to have a very high affinity for water. Kaczmarek [54] produced a sodium alginate film enriched with tannic acid and obtained results that, in 10 s, were similar to those obtained in this study, confirming that despite the decrease in the hygroscopy of the material, the hydrophilic nature of the film remained. This behavior was observed by Lu et al. [12], who analyzed the incorporation of peach peel powder into sodium alginate films with glycerol. The addition of the powder resulted in a significant increase in the hydrophobicity of the films, but the films remained hydrophilic, which was attributed to the greater surface roughness induced by the material. This additional roughness contributed to a reduction in the surface energy, which in turn promoted a slight increase in the level of hydrophobicity [55].

All the bioplastic films, with or without OBP, were completely soluble in water after 10 min. Therefore, solubility and swelling analyses could not be performed according to the article’s methodology within the stipulated time (Table 1). Sucheta et al. [17] observed a similar effect in films produced with alginate, pectin, and orange peel, in which 98.1% of the film was solubilized after 24h in water. Cabello et al. [30] evaluated pectin films with different glycerol contents. The authors reported that higher amounts of glycerol added to the film resulted in more hydrophilic materials because glycerol alters the molecular interconnectivity between the pectin chains and thus allows water molecules to be absorbed, resulting in the complete solubility of the films. Deng et al. [56] also produced sodium alginate films, with or without gluconolactone and whey protein, and reported that the swelling rate could not be recorded because of the high water solubility and miscibility of sodium alginate films.

### 3.9. Scanning Electron Microscopy (SEM)

**Figure 3:**
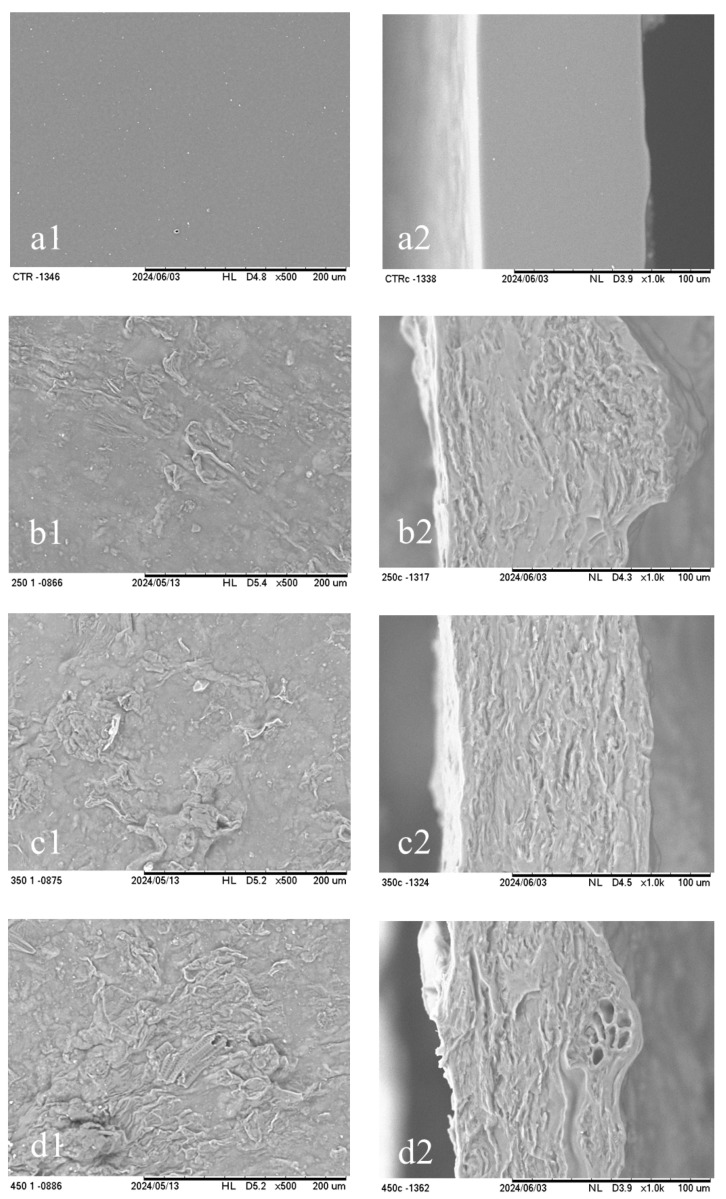
SEM micrographs of the (**1**) cross-section (1000×) and the (**2**) surface (500×) of alginate-based films with different concentrations of OBP (orange by-product): (**a**) 0-OBP (0 g of OBP), (**b**) 25-OBP (2.5 g of OBP), (**c**) 35-OBP (3.5 g of OBP), and (**d**) 45-OBP (4.5 g of OBP).

Figure 3 shows the morphologies of the SA-based films. The surfaces of the control films without OBP (0-OBP) and the cross-section exhibited a smooth, homogeneous, and compact area without phase separation, showing complete miscibility and compatibility of the elements in the matrix film formation, as shown in Figure 3(a1,a2).

Deng et al. [56] reported a similar morphology for sodium alginate films. In contrast, the SA-based films reinforced with OBP showed rougher, more heterogeneous, and irregular areas. The SEM surface images confirm the contact angle analysis, which demonstrates that this roughness contributes to hydrophobicity, while the irregularities can be attributed to the presence of fibers in the OBP. Yaradoddi et al. [57] produced films from solid orange peel waste with glycerol, with a similar surface, and reported that the films had a rougher surface owing to the presence of different fiber shapes and lignocellulosic materials. The same irregularity was observed in the cross-section, which presented regions of vascular bundles of the citrus fruit, as demonstrated by Terzioglu et al. [31]. This analysis corroborates the results obtained for the mechanical properties presented in this study, indicating the concentration of fibers inside the film. Merino et al. [58] prepared a film using pectin and avocado waste (seeds and peels). From the surface and cross-sectional SEM images, they observed low cohesion and a porous structure similar to that found in this study. Therefore, it could be instead that organic materials strengthened the structure of natural polymer films.

### 3.10. Thermogravimetry Analysis (TGA)

**Figure 4:**
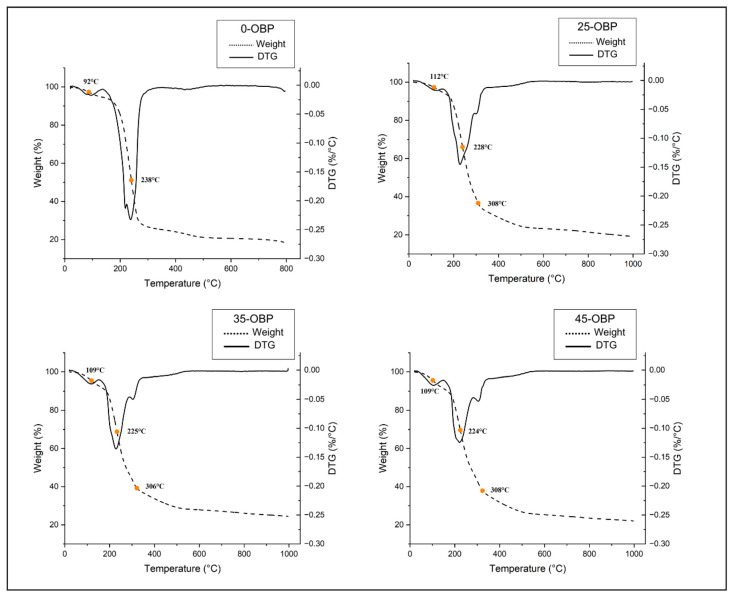
Thermogravimetry analysis of sodium alginate-based films reinforced with different concentrations of OBP (orange by-product): 0-OBP (0 g OBP); 25-OBP (2.5 g OBP); 35-OBP (3.5 g OBP); and 45-OBP (4.5 g OBP).

TGA was performed to determine the thermal stabilities of the formulated bioplastic samples. Two stages of decomposition were observed in the SA-based film (0-OBP) (Figure 4).

Derivative thermogravimetric (DTG) analysis was performed to determine the degradation temperatures of the films. The first mass loss (Tonset) for the 0-OBP film was observed around 92 °C, associated with water volatilization (Figure 4). In contrast, the films containing 25-OBP to 45-OBP demonstrated greater thermal resistance, with the first mass loss occurring between 109 and 112 °C. This temperature increase is attributed to the presence of less water in these films, as indicated by the water content analysis. Since 0-OBP has a higher water content, evaporation occurs more easily than in films with OBP, where water is more strongly bound to the alginate–glycerol matrix [48].

The decomposition temperatures (Td) were similar between the films, approximately 224–238 °C for both 0-OBP and OBP films. However, in films with OBP, a third peak was observed, related to the degradation of monosaccharide rings, occurring between 306 and 308 °C.

The mass loss in these decomposition stages was greater in films without OBP because of the pyrolysis of sodium alginate, which occurs at approximately 238 °C and results in the breakdown of adjacent hydroxyl groups, releasing water molecules and breaking the ether bonds of the structure [59]. In films with OBP, the mass loss was lower, probably due to hydrogen bonds between OBP, alginate, and glycerol, as demonstrated by the FTIR analysis.

In the final stage, the films underwent decarboxylation, forming carbon residues between 550 °C and 790 °C [60]. Similar results were obtained by Barone et al. [61], who developed antioxidant and aromatic films based on persimmons with orange peel. In another study, Meydanju, Pirsa, and Farzi [62] identified two stages in the thermal analysis of lemon powder residues with pectin and TiO_2_ nanoparticles, the latter of which involved the loss of volatile compounds at approximately 150 °C.

## 4. Conclusions

The application of orange by-product powder (OBP) as a reinforcing agent in sodium alginate films was demonstrated in accordance with the precepts of the circular economy. The results revealed that the incorporation of OBP significantly improved the thermal, structural, and mechanical properties of the films. In particular, the addition of OBP increased the mechanical strength and Young’s modulus. In addition, OBP helped reduce the water content and increase the contact angle, facilitating the cross-linking of the matrices and improving the hydrophobicity of the material without compromising its hydrophilic character. This study contributes to the development of new sustainable materials and presents a new circular economy route for the underutilized product of orange juice extraction. Future studies are needed to improve the hydrophilicity of the polymer matrix and explore its applications as a packaging material. Therefore, this study showed that OBP represents a promising approach as a reinforcing component for sodium alginate films, contributing to renewable and sustainable plastic packaging and reducing environmental pollution.

## Figures and Tables

**Table 1:** Properties of sodium alginate-based films reinforced with different concentrations of OBP (orange by-product): 0-OBP (0 g of OBP); 25-OBP (2.5 g of OBP); 35-OBP (3.5 g of OBP); and 45-OBP (4.5 g of OBP).

Parameters	Bioplastic Formulation			
	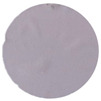 0-OBP	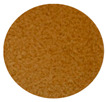 25-OBP	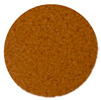 35-OBP	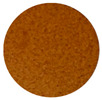 45-OBP
Thickness (mm)	0.02 ± 0.01 ^a^	0.07 ± 0.009 ^b^	0.08 ± 0.02 ^b^	0.08 ± 0.01 ^b^
L*	97.73 ± 1.11 ^a^	75.72 ± 0.29 ^b^	72.61 ± 1.01 ^c^	70.72 ± 1.18 ^d^
a*	−0.02 ± 0.24 ^a^	6.39 ± 0.11 ^b^	8.00 ± 0.56 ^c^	9.18 ± 0.81 ^d^
b*	−4.33 ± 0.79 ^a^	36.25 ± 1.94 ^b^	39.03 ± 2.81 ^b^	40.61 ± 3.38 ^c^
ΔE	-	46.87 ± 1.59 ^a^	51.02 ± 2.59 ^b^	53.53 ± 2.85 ^b^
Water content (%)	37.75 ± 5.80 ^a^	27.33 ± 4.28 ^b^	26.46 ± 1.76 ^b^	24.49 ± 1.47 ^b^
Transparency (%)	22.61 ± 2.05 ^a^	19.03 ± 0.11 ^b^	17.34 ± 0.33 ^b^	18.28 ± 0.44 ^b^
TS (MPa)	7.99 ± 0.91 ^a^	10.92 ± 1.25 ^b^	15.11 ± 0.46 ^c^	18.00 ± 1.38 ^d^
EB (%)	64.86 ± 8.99 ^a^	26.33 ± 3.95 ^b^	21.76 ± 3.98 ^b^	22.88 ± 2.06 ^b^
YM (MPa/%)	0.13 ± 0.03 ^a^	0.39 ± 0.07 ^b^	0.78 ± 0.28 ^c^	0.95 ± 0.22 ^c^
Contact angle (°)	57.60 ± 0.41 ^a^	65.12 ± 2.44 ^b^	70.07 ± 1.48 ^c^	70.34 ± 0.98 ^c^
Swelling	ND	ND	ND	ND
Solubility	ND	ND	ND	ND

Note: Means followed by different lowercase letters in the same line represent statistical differences between formulations according to Tukey’s test (*p* < 0.05).

## Data Availability

The original contributions presented in this study are included in the article/Supplementary Material. Further inquiries can be directed to the corresponding author.

## Supplementary Materials

The following supporting information can be downloaded at: https://www.mdpi.com/article/10.3390/polym16233382/s1, Figure S1: Effect of sodium alginate concentration on (A) elongation and (B) tensile strength of films containing 5 g of glycerol and 2.5 g of OBP (orange by-product): 05-SA (0.5 g sodium alginate), 10-SA (1.0 g sodium alginate), 15-SA (1.5 g sodium alginate), 20-SA (2 g sodium alginate) and 20-SA (2 g sodium alginate); Figure S2: Effect of glycerol concentration on (A) elongation and (B) tensile strength of films containing 2.5 g of sodium alginate and 2.5 g of OBP (orange by-product): 10-G (10% of the total dry mass), 15-G (15% of the total dry mass), 17.5-G (17.5% of the total dry mass), and 20-G (20% of the total dry mass); Figure S3: Deconvolution of the FT-IR signal in the -OH group region of the control, 25-OBP, 35-OBP and 45-OBP films.
