# Supplementary material for: Development of Sodium Alginate Bioplastic Reinforced with Dried Orange Juice By-Product for Use in Packaging
Source: Polymers (Basel). 2024 Nov 30;16(23):3382. doi: 10.3390/polym16233382 (PMC11644364; doi:10.3390/polym16233382)
Supplement: Supplementary file 1 [file polymers-16-03382-s001.zip › polymers-3272672-supplementary.pdf]

Supplementary Material

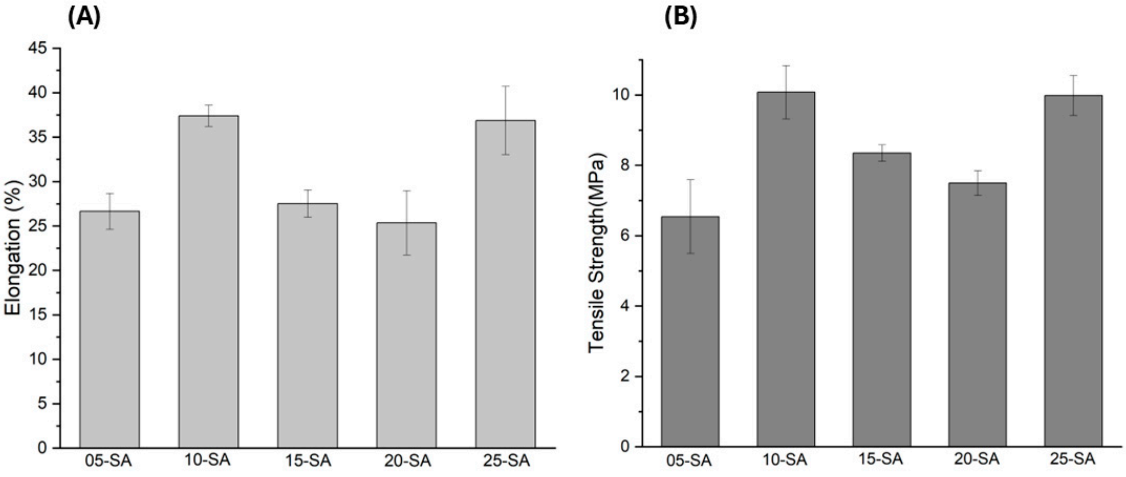

Figure S1: Effect of sodium alginate concentration on (A) elongation and (B) tensile strength of films containing 5 g of glycerol and 2.5 g of OBP (orange by-product): 05-SA (0.5g sodium alginate), 10-SA (1.0g sodium alginate), 15-SA (1.5g sodium alginate), 20-SA (2g sodium alginate) and 20-SA (2g sodium alginate).

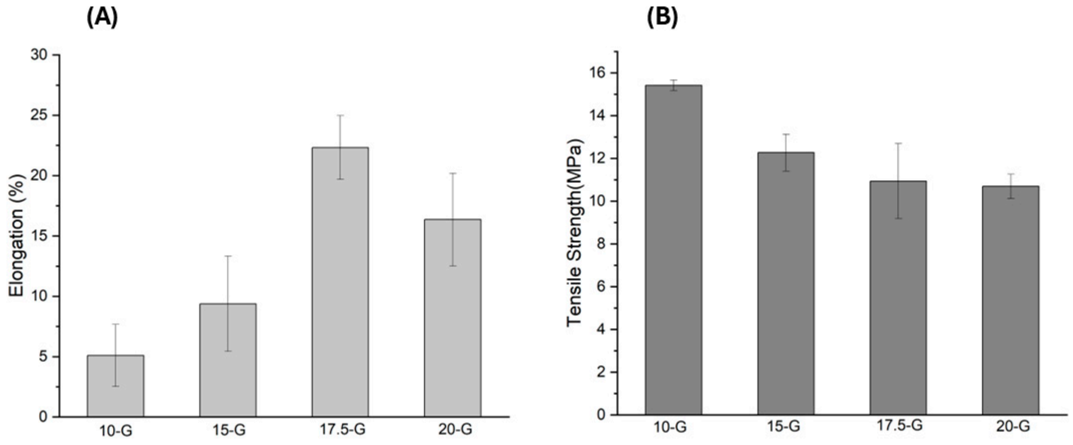

Figure S2: Effect of glycerol concentration on (A) elongation and (B) tensile strength of films containing 2.5 g of sodium alginate and 2.5 g of OBP (orange by-product): 10-G (10% of the total dry mass), 15-G (15% of the total dry mass), 17.5-G (17.5% of the total dry mass), and 20-G (20% of the total dry mass).

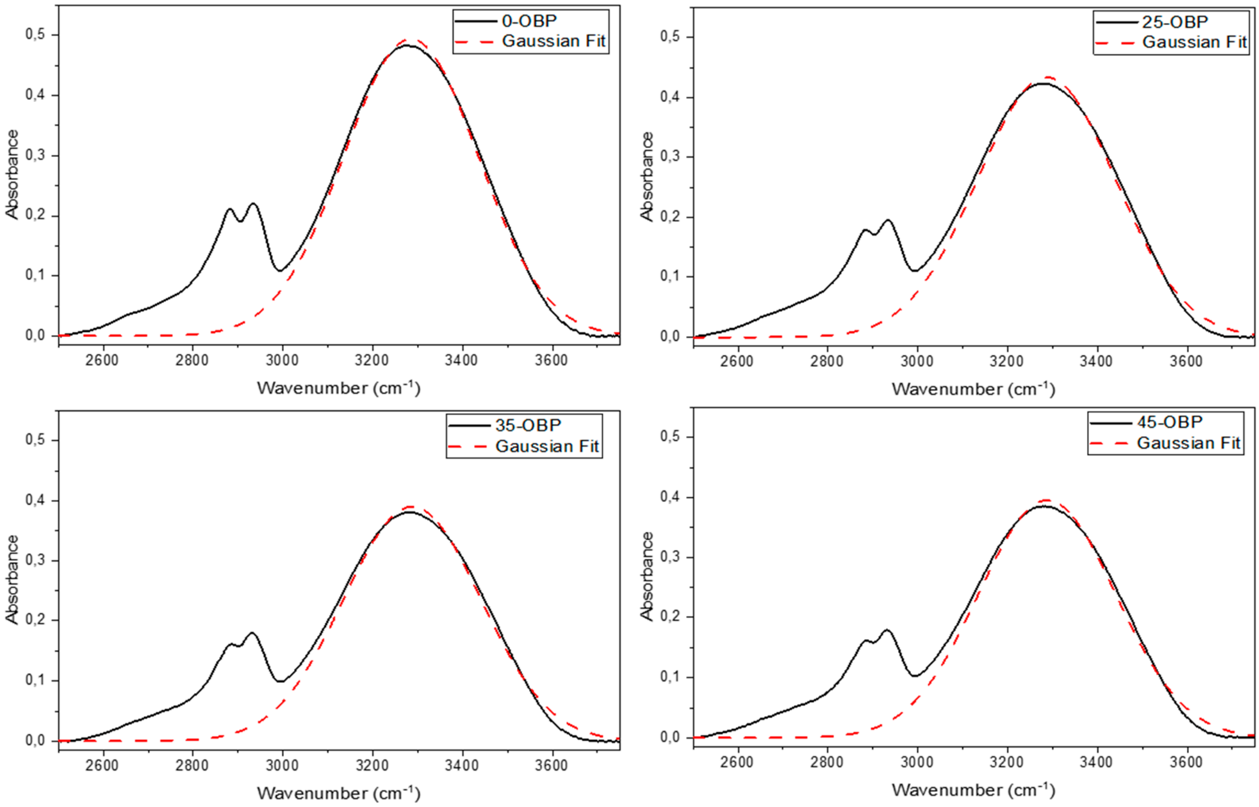

Figure S3: Deconvolution of the FT-IR signal in the -OH group region of the control, 25-OBP, 35-OBP and 45-OBP films.
